# How a needs assessment study taught us a lesson about the ethics of educational research

**DOI:** 10.1007/s40037-017-0356-y

**Published:** 2017-05-30

**Authors:** Lara Varpio, Anne McCarthy

**Affiliations:** 1 Department of Medicine, Uniformed Services University of the Health Sciences, Bethesda, MD USA; 2 Department of Medicine, University of Ottawa, Ottawa, Ontario Canada

**Keywords:** Ethics, Global health, Needs assessment, Safety

## The story

Nearly 10 years ago, in response to a surge of learner interest and participation in global health electives (GHEs) [1], we began collaborating on a qualitative needs assessment to inform the development of GHE-related educational materials for our institution’s medical learners (at the time, we both worked at the University of Ottawa, Canada) [2, 3]. During that needs assessment work, we sought out local research participants who had previous GHE experience – either as trainees or as faculty members. We collected data through individual semi-structured interviews, using a series of open-ended questions designed to encourage participant reflection on the knowledge, skills, and attitudes they needed to successfully participate in the GHE [2, 3]. We submitted the interview protocol as part of our submission to the university’s research ethics board which approved the study. As part of that interview protocol, we asked participants to describe particularly memorable GHE-related stories, and to recount GHE events that they felt they were unprepared to contend with.

With these prompts, we were expecting to collect data from which to construct themes relating to the lessons learned through GHE participant experience. We anticipated developing pre-departure training that could better prepare our learners for the unique challenges that GHEs pose. In essence, we expected to collect ‘if I knew then what I know now’ kinds of tips and advice that could inform the construction of a GHE pre-departure educational innovation.

Participants shared valuable insights on a number of topics including navigating the cultural differences that separated their Western expectations from those of the low-resource hosting institutions, and the importance of building trusting relationships with the care providers in the hosting community [2, 3]. But they also shared stories we were not anticipating.

## Surprising outcomes

In some of these interviews, participants recounted stories of physical, sexual, and emotional trauma. We heard stories of needle stick injuries and the subsequent fear of HIV infection, without ready access to post-exposure prophylaxis. We heard stories of hiding from armed gangs looking for tourists. We heard stories of being sexually assaulted in public spaces. We heard stories of emotional anguish experienced from treating children for diseases that were death sentences in the host country, but often times quite easily cured in Western nations. We heard stories of learners being pressured to provide unsupervised patient care that exceeded their scope of training.

We were not expecting to hear such stories.

Worse – we weren’t prepared to hear such stories.

Realizing the seriousness of the stories we were hearing, we immediately stopped the study and found counselling support for all the study’s participants. We contacted the research ethics board and informed them of the narratives our participants were sharing. We informed the medical school’s leadership of these stories and of the need to support students in dealing with the outcomes of GHE engagement – outcomes that ranged from the jubilation of supporting a community in need, to the deep and personal trauma of sexual assault. The medical school instituted a Global Health Office. Its purpose was to ensure that the GHEs that our learners were participating in were institutionally observed, had appropriate expectations, and ideally involved formal, long-term partnerships with host countries. The office staff worked to ensure the safety of the learners who participated in these GHEs. They also worked to protect the interests and safety of the community members in the host countries.

With safety protocols and debriefing structures in place for our research participants, the research ethics board again reviewed and approved our study. We resumed and eventually completed this research [2, 3]. In fact, this study spurred us to intentionally explore the ethical and safety risks of GHEs to learners [4]. These data were essential to the institution so that it could responsibly prepare and support learners who wanted to participate in GHEs. The institution financially backed our continued research and the development of the Global Health Office. From then on, any learner who participated in a GHE attended pre-departure training. There were also measures in place to support learners while they were away on GHEs. And there were individual and group debriefing sessions awaiting learners upon their return from GHEs.

## Lessons learned

Obviously an important lesson that we learned from this experience was the need for careful management and implementation of GHE experiences for learners. While GHEs have been established as highly valuable experiences for medical learners [5], these benefits cannot be reaped if we do not ensure the safety of the participating learners and benefits to the hosting communities.

But there is another lesson to be learned here – one related to the ethics of educational research.

All too often, when we submit our educational research projects to our local research ethics board, it seems a pro forma action – something we do because it is expected, because we need its approval to write one line in a manuscript, because it is a formality we must respect. And the ethics forms that educational researchers fill out – forms that are usually designed with clinical research in mind – often have little relevance for the research we are proposing. So we follow procedure. We thoroughly describe the project’s procedures in the ethics review submission. We explain why there is minimal risk of harm to the research participants. We confirm that we will collect informed consent from our participants. We report how we will store the data securely.

But this interviewing experience taught us the important difference between *procedural ethics* and *ethics in practice* [6]. While procedural ethics involves obtaining approval from the research ethics board to engage in research that involves humans, ethics in practice are the ‘ethical issues that arise in the doing of research’ [6, p. 263]. As educational researchers, we have a responsibility to adhere to the highest standards of procedural ethics. But we must also simultaneously be vigilant about continuously and reflexively considering the day-to-day, unplanned moments in our study that require us to demonstrate ethics in practice.

Our experiences with the GHE interviews made us keenly aware that ethical research requires that we constantly monitor ourselves and our research findings to recognize when an ethical consideration is being threatened and to respond appropriately. Although our research had not put participants at risk, our investigation had uncovered an ethical issue that our institution was not aware of and that needed to be addressed.

Listening to these participants’ stories was a shocking and emotional experience for us. We had no idea these GHE-related events were happening. We realized that our research study needed to immediately morph into a crisis management and support-oriented intervention. Obviously these stories continue to haunt us; here we are writing about them nearly 10 years later. But we firmly believe that this experience has made us better researchers.

We have changed procedures in our research endeavours to ensure that ethics in practice is always at the fore of our thinking. Part of the training we give to our research assistants now involves training them to engage responsibly in ethics in practice. Before we engage in any data collection, we now make sure to know which support services are available to our participant populations at the time of data collection in case those services are needed. We are also developing a post-participation handout that can be customized for each project we undertake that lists the support services available to participants, and the contact information for the study’s principal investigator. It is now part of *our own procedural ethics* to include these kinds of *ethics in practice* considerations and preparations in our research ethics board submissions.

We are confident that we reacted ethically and appropriately when we heard the stories of trauma from our GHE participants. But that experience taught us a valuable lesson. We are now better prepared for such situations and we proactively plan how to manage the ethics in practice that are part of every research project.

## Moral of the story

While obtaining ethics board approval may seem to be a formal hurdle to be jumped, it is vitally important that we remember that all studies carry risks for the participants and have the potential to unearth yet-to-be-identified problems. As researchers, we must be prepared to attend to ethics in practice because we don’t know when unexpected ethical challenges will arise.
